# Effects of gastroesophageal reflux disease treatment with proton pump inhibitors on the risk of acute exacerbation and pneumonia in patients with COPD

**DOI:** 10.1186/s12931-023-02345-1

**Published:** 2023-03-11

**Authors:** Jieun Kang, Rugyeom Lee, Sei Won Lee

**Affiliations:** 1 Division of Pulmonary and Critical Care Medicine, Department of Internal Medicine, Ilsan Paik Hospital, Inje University College of Medicine, Goyang, Republic of Korea; 2 Department of Preventive Medicine, School of Medicine, Kyung Hee University, Seoul, Republic of Korea; 3 Artificial Intelligence and Big-Data Convergence Center, Gachon University Gil Medical Center, Gachon University College of Medicine, Incheon, Republic of Korea; 4 Department of Pulmonary and Critical Care Medicine, Asan Medical Center, University of Ulsan College of Medicine, 88 Olympic-ro 43-gil, Songpa-gu, Seoul, 05505 Republic of Korea

**Keywords:** Chronic obstructive pulmonary disease, Gastroesophageal reflux disease, Proton pump inhibitor, Acute exacerbation, Pneumonia

## Abstract

**Background:**

Gastroesophageal reflux disease (GERD) has been suggested as a risk factor for acute exacerbation of chronic obstructive pulmonary disease (COPD). However, it remains undetermined whether proton pump inhibitor (PPI) treatment reduces the risk of exacerbation or affects the risk of pneumonia. This study aimed to evaluate the risks of both exacerbation and pneumonia following PPI treatment for GERD in patients with COPD.

**Methods:**

This study used a reimbursement database of the Republic of Korea. Patients aged ≥ 40 years with COPD as a main diagnosis and who received PPI treatment for GERD at least for 14 consecutive days between January 2013 and December 2018 were included in the study. A self-controlled case series analysis was conducted to calculate the risk of moderate and severe exacerbation and pneumonia.

**Results:**

A total of 104,439 patients with prevalent COPD received PPI treatment for GERD. The risk of moderate exacerbation was significantly lower during the PPI treatment than at baseline. The risk of severe exacerbation increased during the PPI treatment but significantly decreased in the post-treatment period. Pneumonia risk was not significantly increased during the PPI treatment. The results were similar in patients with incident COPD.

**Conclusions:**

The risk of exacerbation was significantly reduced after PPI treatment compared with the non-treated period. Severe exacerbation may increase due to uncontrolled GERD but subsequently decrease following PPI treatment. There was no evidence of an increased risk of pneumonia.

**Supplementary Information:**

The online version contains supplementary material available at 10.1186/s12931-023-02345-1.

## Background

Chronic obstructive pulmonary disease (COPD) is one of the major causes of morbidity and mortality worldwide [1, 2]. COPD often coexists with other diseases that may significantly influence the prognosis [2]. Gastroesophageal reflux disease (GERD) is one of the common comorbidities of COPD [3–5], characterized by abnormal reflux of acidic gastric contents into the esophagus, which results in reflux symptoms and damage to the esophageal mucosa [6]. Previous studies have shown that patients with coexistent GERD and COPD have worse quality of life and more severe shortness of breath than those with COPD but without GERD [7, 8]. More importantly, GERD has been suggested as a risk factor for acute exacerbation [7, 9–13]. In a few studies, the severity of reflux, which was determined by a questionnaire score or esophageal pH monitoring, was correlated with a greater exacerbation risk [14, 15].

One may expect that appropriate treatment of GERD can reduce the risk of exacerbation in patients with COPD. However, consistent evidence to support this hypothesis is lacking. Few observational studies have reported that treatment with anti-acid agents, such as proton pump inhibitors (PPIs), was associated with a lower risk of exacerbation [7, 16], while other studies have found that patients receiving acid-suppressive treatment remain at a high risk of exacerbation [8, 11, 17]. To date, only one randomized controlled trial was designed to evaluate the effect of PPI therapy in preventing exacerbation; however, it excluded patients with GERD [18]. Therefore, it remains undetermined if GERD treatment has a role in reducing the risk of acute exacerbation.

Another consideration related to GERD treatment is that there has been a concern for an increased risk of pneumonia with PPI use. Several studies suggested a positive association between PPIs and community-acquired pneumonia [19–23]. Administration of PPIs potently decreases gastric acid secretion and may lead to insufficient clearance and even overgrowth of ingested bacteria [24]. Given that pneumonia is another major cause of mortality in patients with COPD [25], the effect of PPIs on pneumonia risk should also be considered in patients with COPD. This study aimed to evaluate the risks of both acute exacerbation and pneumonia following PPI treatment for GERD in patients with COPD.

## Methods

### Data source

This study was performed using a reimbursement database from January 2013 to December 2018 provided by the Health Insurance Review and Assessment Service of the Republic of Korea. The Health Insurance Review and Assessment Service is a government-affiliated agency that evaluates the accuracy of claims for National Health Insurance, a compulsory insurance system that covers 96.6% of the total South Korean population of 48.6 million, and National Medical Aid covering the remaining 3.5%. The database contains diagnoses, prescribed medications, medical procedures, information on visits to outpatient clinic or emergency department and hospital admissions, and payment of health insurance that can estimate the patient’s level of income. Several studies have been published using the Health Insurance Review and Assessment Service database. This study protocol was approved by the Institutional Review Board of the Asan Medical Center, Seoul, South Korea (IRB No. 2020-0218). The need for informed consent was waived because this study used existing database provided in a de-identified format.

### Study subjects

We included adult patients aged ≥ 40 years with COPD as a main diagnosis and who received PPI treatment at least for 14 consecutive days for GERD from January 2013 to December 2018. COPD was defined using the following criteria: (1) International Classification of Diseases (ICD)-10 codes J42.x, J43.x, and J44.x and 2) prescription of COPD medications, including long-acting bronchodilators at least twice a year. The types of COPD medications and PPIs that were used to define study patients are presented in Additional file 1: Tables S1 and S2, respectively. Patients were excluded if they met any of the following criteria: (1) death during the study period; (2) prescription of corticosteroid for ≥ 60 days within 1 year before the PPI treatment; (3) PPI treatment without ICD-10 codes for GERD (K21, K21.0, or K21.9); (4) PPI treatment during hospitalization; (5) previous PPI treatment within 90 days before the study period; and (6) PPI treatment starting concurrently with corticosteroid or antibiotics.

Patients were further categorized into prevalent or incident COPD. Patients with prevalent COPD were as study participants who met the abovementioned criteria at the time of study initiation (total study population). Patients with incident COPD were a subgroup of patients who did not have a COPD diagnosis code in the previous year (newly diagnosed COPD).

### Study design

**Fig. 1:**
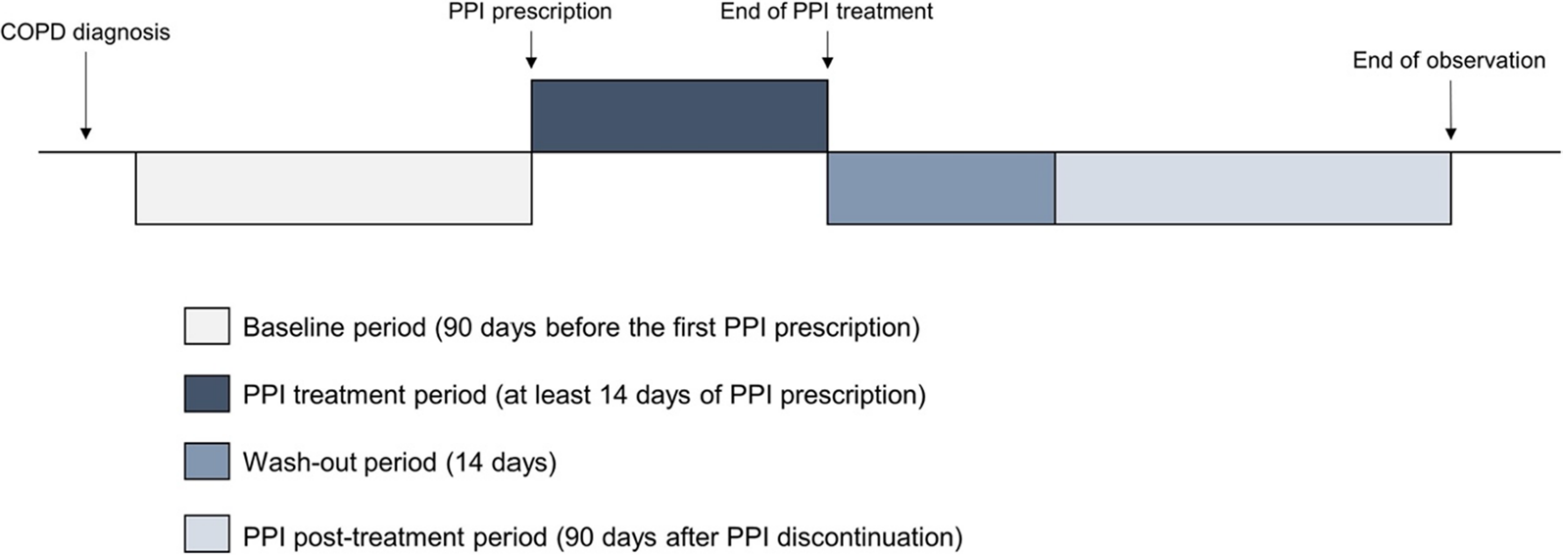
Diagram representing the self-controlled case series design of the study

We conducted a self-controlled case series analysis in which each study subject acted as their own control, to minimize the influence of individual risk factors for acute exacerbation or pneumonia that individual subjects have. Figure 1 shows the design of this study. Index date was defined as the initiation date of PPI with ICD-10 codes for GERD. Treatment and baseline periods were defined as the 90 consecutive days following and before the index date, respectively. In patients in whom the observation period was adequately long, the 90 consecutive days after 14 days of washout from the last day of PPI treatment was defined as the post-treatment period.

### Outcomes

The co-primary outcomes were risks of acute exacerbation (moderate and severe) and pneumonia. Moderate exacerbation was defined as an outpatient clinic visit with an ICD-10 code of COPD and prescription of systemic steroid (prednisolone 20 mg or an equivalent dose) for at least 3 days with or without antibiotics. Severe exacerbation was defined as hospitalization or an emergency department visit with an ICD-10 code of COPD and prescription of systemic steroid (prednisolone 20 mg or an equivalent dose) for at least 3 days with or without antibiotics. Pneumonia was defined as an outpatient clinic or emergency department visit, or hospitalization with an ICD-10 code of pneumonia (J11.x–J18.x) accompanying chest radiography and antibiotic prescription for at least 3 days. The identification codes for steroids and antibiotics used in this study are listed in Additional file 1: Tables S3 and S4, respectively.

### Statistical analysis

Data were presented as numbers with percentages for categorical variables. The covariates selected included age at the index date, sex, presence of comorbidities, and the estimated income level. Comorbidities were identified by ICD-10 codes, all of which were assessed 180 days prior to the index date. Comorbidities that might influence the risk for exacerbation and pneumonia were selected based on the Charlson Comorbidity Index and were added to the known risk factors.

Study patients were classified as population 1 or 2 according to the presence of the observation period after PPI discontinuation (post-treatment period). In population 1, the risks of moderate exacerbation, severe exacerbation, and pneumonia in the treatment period were calculated as incident rate ratios (IRRs) using the baseline period as a reference. In population 2, in addition to the risks during the treatment period, IRRs of moderate and severe exacerbation and pneumonia in the post-treatment periods were calculated. Survival curves were constructed using Kaplan–Meier estimates and compared with the use of the log-rank test. All statistical analyses were performed using the SAS Enterprise Guide software (version 6.1, SAS Institute, Inc., Cary, NC, USA).

## Results

### Baseline characteristics

A total of 104,439 patients with prevalent COPD were identified with PPI treatment for GERD (population 1). Among them, 54,689 patients had an observation period of 90 days after PPI discontinuation (population 2). Table 1 shows the baseline characteristics of populations 1 and 2. In population 1, men accounted for 54.6%. Patients aged 70–79 years were the most common followed by those aged 60–69 years. Long-acting beta-2 agonist/inhaled corticosteroids were the most frequently prescribed inhaler (17.4%), followed by long-acting muscarinic antagonists (11.7%). Patients had frequent comorbidities, such as asthma, hyperlipidemia, hypertension, and diabetes. In population 2, the baseline characteristics were similar to that of population 1.

**Table 1:** Baseline characteristics of patients with prevalent chronic obstructive pulmonary disease

	Population 1	Population 2
	104,439	54,689
Male	57,030 (54.6)	30,065 (55.0)
Age (years)		
40–49	4638 (4.4)	3015 (5.5)
50–59	17,527 (16.8)	10,185 (18.6)
60–69	33,079 (31.7)	17,606 (32.2)
70–79	35,869 (34.3)	17,867 (32.7)
≥ 80	13,326 (12.8)	6016 (11.0)
COPD medication		
LAMA	12,266 (11.7)	6277 (11.5)
LABA	3187 (3.1)	1650 (3.0)
LAMA/LABA	2869 (2.7)	1241 (2.3)
LABA/ICS	18,222 (17.4)	9259 (16.9)
LAMA/LABA/ICS	4211 (4.0)	2186 (4.0)
Methylxanthines	49,002 (46.9)	25,363 (46.4)
Others	36,920 (35.4)	19,048 (34.8)
Comorbidity		
Asthma	80,909 (77.5)	41,482 (75.9)
Hyperlipidemia	72,633 (69.5)	35,569 (65.0)
Hypertension	64,886 (62.1)	31,419 (57.5)
Diabetes	51,598 (49.4)	24,624 (45.0)
Cerebrovascular disease	23,984 (23.0)	10,634 (19.4)
Cancer	20,228 (19.4)	9584 (17.5)
Congestive heart failure	18,720 (17.9)	7957 (14.5)
Arrhythmia	12,669 (12.1)	5828 (10.7)
Bronchiectasis	12,513 (12.0)	6141 (11.2)
Renal disease	4506 (4.3)	1915 (3.5)
Valvular disease	1998 (1.9)	860 (1.6)
Environmental lung disease	1164 (1.1)	541 (1.0)
Liver disease	896 (0.9)	386 (0.7)
Quintiles for estimated household income		
1	11,247 (10.8)	4775 (8.7)
2	17,367 (16.6)	9148 (16.7)
3	16,227 (15.5)	8731 (16.0)
4	23,034 (22.1)	12,450 (22.8)
5	36,564 (35.0)	19,585 (35.8)

Data are presented as numbers (%)

*COPD* chronic obstructive pulmonary disease, *LABA* long-acting beta-2 agonist, *LAMA* long-acting muscarinic antagonist, *ICS* inhaled corticosteroid

### Risks of acute exacerbation and pneumonia

**Fig. 2:**
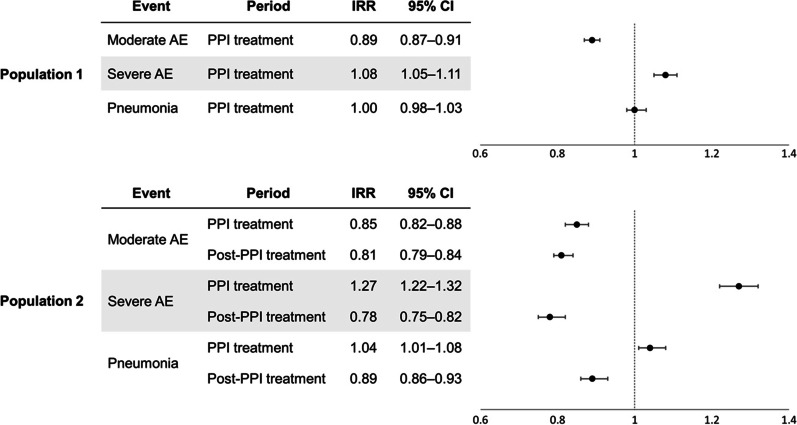
Risk of study outcomes in patients with prevalent chronic obstructive pulmonary disease. In population 1, the IRRs of moderate and severe exacerbation and pneumonia during the PPI treatment period were calculated, compared to that at the baseline. Population 2 comprised patients with an observation period of at least 90 days after PPI discontinuation, and the IRRs of moderate and severe exacerbation and pneumonia during the PPI treatment and post-treatment periods were calculated. *AE* acute exacerbation, *IRR* incidence rate ratio, *CI* confidence interval, *PPI* proton pump inhibitor

Figure 2 shows the risk of study outcomes in patients with prevalent COPD. In population 1, the risk of moderate exacerbation was significantly lower during the PPI treatment period (IRR, 0.89), whereas those of severe exacerbation was higher (IRR, 1.08). The risk of pneumonia was not significantly increased compared to that at the baseline period.

In population 2, the risks of study outcomes during the PPI treatment and post-treatment periods were compared to those during the baseline period. The risk of moderate exacerbation was significantly lower in both the treatment and post-treatment periods (IRR, 0.85 and 0.81, respectively). Although the risks of severe exacerbation and pneumonia significantly increased during the PPI treatment period (IRR, 1.27 and 1.04, respectively), they decreased during the post-treatment period (IRR, 0.78 and 0.89, respectively).

### Subgroup analysis: incident COPD cases

A total of 20,704 patients had new COPD diagnosis during the study period and were treated with PPI for GERD (population 1). Among them, 10,988 patients had an observation period of 90 days after PPI discontinuation (population 2). The baseline characteristics are shown in Table 2.

**Table 2:** Baseline characteristics of patients with incident chronic obstructive pulmonary disease

	Population 1	Population 2
	20,704	10,988
Male	11,134 (53.8)	5984 (54.5)
Age (years)		
40–49	1070 (5.2)	700 (6.4)
50–59	3761 (18.2)	2185 (19.9)
60–69	6811 (32.9)	3642 (33.1)
70–79	6697 (32.3)	3352 (30.5)
≥ 80	2229 (10.8)	1109 (10.1)
COPD medication		
LAMA	2429 (11.7)	1251 (11.4)
LABA	749 (3.6)	402 (3.7)
LAMA/LABA	1050 (5.1)	494 (4.5)
LABA/ICS	4891 (23.6)	2523 (23.0)
LAMA/LABA/ICS	604 (2.9)	331 (3.0)
Methylxanthines	13,400 (64.7)	7063 (64.3)
Others	10,217 (49.3)	5336 (48.6)
Comorbidity		
Asthma	16,438 (79.4)	8574 (78.0)
Hyperlipidemia	14,752 (71.3)	7345 (66.8)
Hypertension	12,483 (60.3)	6143 (55.9)
Diabetes	10,171 (49.1)	4970 (45.2)
Cerebrovascular disease	4502 (21.7)	2064 (18.8)
Cancer	3855 (18.6)	1897 (17.3)
Congestive heart failure	3597 (17.4)	1629 (14.8)
Arrhythmia	2515 (12.1)	1164 (10.6)
Bronchiectasis	2089 (10.1)	1069 (9.7)
Renal disease	870 (4.2)	391 (3.6)
Valvular disease	353 (1.7)	147 (1.3)
Environmental lung disease	182 (0.9)	84 (0.8)
Liver disease	179 (0.9)	94 (0.9)
Quintiles for estimated household income		
1	1972 (9.5)	834 (7.6)
2	3457 (16.7)	1862 (16.9)
3	3369 (16.3)	1832 (16.7)
4	4724 (22.8)	2590 (23.6)
5	7182 (34.7)	3870 (35.2)

Data are presented as numbers (%)

*COPD* chronic obstructive pulmonary disease, *LABA* long-acting beta-2 agonist, *LAMA* long-acting muscarinic antagonist, *ICS* inhaled corticosteroid

**Fig. 3:**
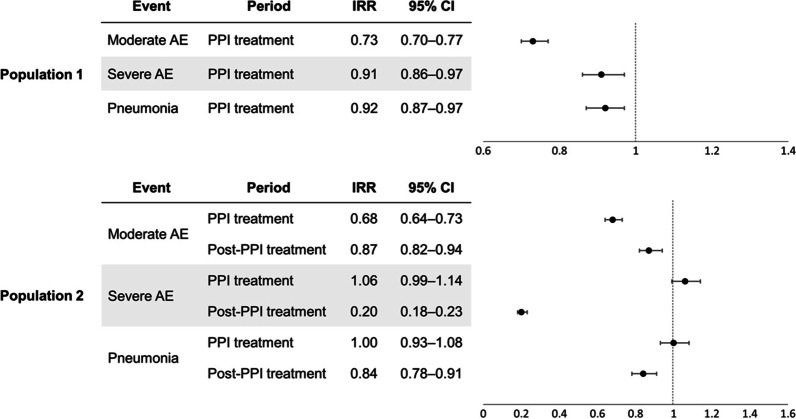
Incidence rate ratios of study outcomes in patients with incident chronic obstructive pulmonary disease. In population 1, the IRRs of moderate and severe exacerbation and pneumonia during the PPI treatment period were calculated compared to that at the baseline. Population 2 comprised patients with an observation period of at least 90 days of after PPI discontinuation, and the IRRs of moderate and severe exacerbation and pneumonia during the PPI treatment and post-treatment periods were calculated. *AE* acute exacerbation, *IRR* incidence rate ratio, *CI* confidence interval, *PPI* proton pump inhibitor

In population 1, the risks of moderate and severe exacerbation and pneumonia were significantly lower during the PPI treatment period than that at the baseline period (IRR, 0.73, 0.91, and 0.92, respectively, shown in Fig. 3). In population 2, the risk of moderate exacerbation was significantly lower in the treatment and post-treatment periods (IRR, 0.68 and 0.87, respectively). Although the risks of severe exacerbation and pneumonia in the PPI treatment periods were not significantly different from that at baseline, they were significantly lower in the post-treatment periods (IRR, 0.20 and 0.84, respectively).

## Discussion

In this study, PPI treatment was associated with a decreased risk in moderate exacerbation of COPD. Although severe exacerbation increased during the PPI treatment, the risk became lower than the baseline in the long term, suggesting that the risk of severe exacerbation decreases for GERD with PPI treatment. There was no consistent evidence for an increased risk of pneumonia associated with PPI treatment.

GERD is one of the common comorbidities in patients with COPD and has been suggested as a risk factor of exacerbations. Among previous studies, the study by Hurst et al. on patients from the Evaluation of COPD Longitudinally to Identify Predictive Surrogate End-points (ECLIPSE) cohort found that patients with GERD have an approximately 1.7 times higher risk of exacerbation [26]. However, there is a lack of solid evidence whether GERD treatment reduces the risk of exacerbation. The risk of moderate exacerbation significantly decreased during PPI treatment according to our study results. This risk reduction was maintained when we analyzed patients with an observation period after PPI discontinuation. One possible explanation for the decrease in moderate exacerbation during PPI treatment is that GERD could aggravate cough that might be perceived as an exacerbation episode and this can be effectively prevented by PPI. GERD is one of the common causes of chronic cough [27], and previous studies have shown that patients with GERD report significantly more respiratory symptoms than individuals without GERD [7]. In such cases, the risk of moderate exacerbation can be reduced by PPI treatment because the symptoms rapidly improve with PPI administration.

On the other hand, there was a different trend regarding severe exacerbation. The risk of severe exacerbation seemed to increase during the PPI treatment period; however, the risk was lower after discontinuation of PPI than that at the baseline. The increase in the risk of severe exacerbation may be associated with uncontrolled GERD itself rather than PPI because the risk becomes lower in the post-treatment period than that at the baseline period, indicating that the risk of exacerbation decreases with the PPI effect. The mechanism by which GERD increases the exacerbation risk is thought to be through gastric acid aspiration, which could cause airway inflammation [28]. Even if symptoms resolve after PPI administration, airway inflammation may need time to improve. Thus, there might have been a lag time before the beneficial effects of PPI could be reflected as a reduction of the exacerbation risk. The finding that severe exacerbation risk in the incident COPD cases was lower even during the PPI treatment period than that at baseline also suggests that PPI treatment is associated with a lower risk of exacerbation. In patients with incident COPD, the lag time may have been relatively shorter than in patients with prevalent COPD, leading to a rapid risk reduction effect.

There have been conflicting findings on the impact of PPIs on the risk of exacerbation. In the Copenhagen City Heart Study cohort, Ingebrigtsen et al. found that GERD was associated with an increased risk of COPD exacerbation; however, the risk applied only to those who were not receiving regular antacid treatment [7]. Individuals with GERD regularly treated with antacid medications did not have an increased risk of exacerbation. In contrast, Benson et al. showed that not only the presence of GERD but also the use of PPIs or H2 receptor antagonists was associated with exacerbation [11]. In another prospective observational cohort study, Baumeler et al. reported that patients with GERD receiving PPI treatment remain at a high risk of severe exacerbation [17]. These conflicting results might have been due to the inherent limitations of observational studies. Patients with GERD had more symptoms and poorer health status than those without GERD at baseline [8, 11, 17]. Although baseline differences were adjusted in multivariable analyses, unrecognized confounding factors might have influenced the result of the studies. The nature of our study design—self-controlled case study—minimizes the effect of confounding factors such as genetics, lung function, previous exacerbation history, medication use, and socioeconomic status.

In this study, the risk of pneumonia was also examined. A few previous studies reported an increased risk of pneumonia associated with PPI use [23, 29]. PPIs decrease gastric acidity, which is a defense mechanism of the stomach against ingested pathogens [23], and may predispose a patient to bacterial colonization and overgrowth [30] that can increase the risk of respiratory infections [31]. However, Othman et al. did not find such a relationship in their large population-based study [32]. They examined the risk of community-acquired pneumonia before and after PPI prescription and observed that the incidence rate of pneumonia was higher in the year before PPI prescription than the year after. Likewise, we did not find consistent evidence for an increased risk of pneumonia associated with PPI treatment; instead, a decreased risk was observed with the treatment in several periods. In patients with prevalent COPD, the risk of pneumonia during the PPI treatment period was not significantly different from that at the baseline. Among patients with an observation period extending after PPI discontinuation, the risk of pneumonia increased during PPI treatment and subsequently decreased after PPI discontinuation. In patients with incident COPD, the risk of pneumonia was significantly lower during the PPI treatment and post-treatment periods. Further studies are required to clearly define the association between PPI and pneumonia.

Our study has some limitations. This was a population-based study that identified patients with COPD, acute exacerbation, or pneumonia by operational definitions. The presence of GERD was identified by diagnosis codes and medication prescription without 24-h pH monitoring or esophagogastroduodenoscopy because they are not mandatory in making the diagnosis. It is also possible that patients received PPIs for reasons other than GERD. However, to exclude such a possibility, cases where PPIs were prescribed concurrently with steroids or antibiotics were excluded, and only patients who had been administered PPI for at least 2 weeks were included. Second, detailed information on several risk factors including body mass index and smoking habit was not available. However, the influence of such factors is unlikely because all our comparisons were within each patient. Third, we did not investigate whether different types or doses of PPI affected the risk of acute exacerbation or pneumonia. There are known variations in the acid-suppression potency of different PPI preparations. However, evidence of the superiority of one PPI compared to that of others is limited, and standard-dose PPIs have comparable efficacy to high-dose PPIs in treating GERD symptoms. Therefore, we considered that all PPIs have similar efficacy for treating GERD. Further research is needed to determine whether differences in acid-suppression potency according to PPI types or doses lead to differences in the exacerbation risk. Lastly, most of the included patients were Korean patients. The generalizability of our results to different races or ethnicity should be evaluated in further research.

In conclusion, a significant reduction in the risk of moderate and severe exacerbation was noted after the PPI treatment compared with the non-treated period in patients with COPD with GERD. Although severe exacerbation may increase due to uncontrolled GERD, it subsequently decreases following the PPI treatment. There was no evidence of an increased risk of pneumonia with PPI treatment. GERD is one of the common comorbidities in COPD, and our large-sized real-world study indicated the positive impact of its management on exacerbation.

## Supplementary Information

**Additional file 1: Table S1.** Types of medication used for patients with chronic obstructive pulmonary disease. **Table S2.** Types of proton pump inhibitors. **Table S3.** Types of systemic steroid. **Table S4.** Types of antibiotics.

## Data Availability

The datasets used and analyzed in the current study are from the database of the Korean Health Insurance Review and Assessment Service. Interested researchers may contact globalhira@hira.or.kr for data access requests.

## Abbreviations

COPD: Chronic obstructive pulmonary disease
GERD: Gastroesophageal reflux disease
IRR: Incident rate ratio
PPI: Proton pump inhibitor
