# Supplementary material for: Effects of gastroesophageal reflux disease treatment with proton pump inhibitors on the risk of acute exacerbation and pneumonia in patients with COPD
Source: Respir Res. 2023 Mar 11;24:75. doi: 10.1186/s12931-023-02345-1 (PMC10008570; doi:10.1186/s12931-023-02345-1)
Supplement: Supplementary file 1 — Additional file 1: Table S1. Types of medication used for patients with chronic obstructive pulmonary disease. Table S2. Types of proton pump inhibitors. Table S3. Types of systemic steroid. Table S4. Types of antibiotics. [file 12931_2023_2345_MOESM1_ESM.docx]

**Additional file 1**

**Effects of gastroesophageal reflux disease treatment with proton pump inhibitors on the risk of acute exacerbation and pneumonia in patients with COPD**

Jieun Kang MD, PhD^1*^, Rugyeom Lee PhD^2,3*^ and Sei Won Lee, MD PhD^4^

Table S1. Types of medication used for patients with chronic obstructive pulmonary disease

| Class | Medication | Code |
| --- | --- | --- |
| Short-acting beta-2 agonist (inhaler) | Fenoterol | 157902CLQ |
|  |  | 157903CAE |
|  |  | 157904CAE |
|  |  | 157930CLQ |
|  | Procaterol | 218304CSI |
|  |  | 218330CSI |
|  | Salbutamol | 225502CSI |
|  |  | 225504CSI |
|  |  | 225506CSI |
|  |  | 225508CSI |
|  |  | 225509CSI |
|  |  | 225530CSI |
|  |  | 225531CSI |
|  |  | 225532CSI |
|  | Terbutaline | 235803CSI |
|  |  | 235805CLQ |
|  |  | 235830CLQ |
| Long-acting beta-2 agonist (inhaler) | Formoterol | 163102CSI |
|  |  | 163105CSI |
|  | Indacaterol | 611901CSI |
|  |  | 611902CSI |
|  | Salmeterol | 225801CSI |
|  |  | 225802CSI |
| Systemic beta agonist (oral, intravenous, dermal) | Bambuterol | 113601ATB |
|  |  | 113602ASY |
|  |  | 113801ATB |
|  |  | 113802ATB |
|  |  | 11630ASY |
|  | Clenbuterol | 135301ASY |
|  |  | 135302ATB |
|  |  | 264700ASY |
|  |  | 264800ATB |
|  |  | 135330ASY |
|  |  | 135331ASY |
|  |  | 135332ASY |
|  | Fenoterol | 157901ATB |
|  | Formoterol | 163101ACH |
|  |  | 163101ASY |
|  |  | 163101ATB |
|  |  | 163104ASY |
|  |  | 163104ATB |
|  |  | 163130ASY |
|  |  | 163131ASY |
|  | Procaterol | 218301ATB |
|  |  | 218302ATB |
|  |  | 218303ASY |
|  |  | 374800ASY |
|  | Salbutamol | 225501ATB |
|  |  | 225503ACR |
|  |  | 225503ATB |
|  |  | 225503ATR |
|  |  | 225505ASY |
|  |  | 225507ACR |
|  |  | 225507ATR |
|  |  | 225601ACR |
|  |  | 225601ATR |
|  | Terbutaline | 235801ATB |
|  |  | 235802BIJ |
|  |  | 235803CSI |
|  |  | 235804ASY |
|  |  | 235805CLQ |
|  |  | 235806ATB |
|  |  | 235806ATR |
|  | Tulobuterol | 245701ATB |
| Short-acting muscarinic antagonist | Ipratropium | 177101CLQ |
|  |  | 177103CLQ |
|  |  | 177104CAE |
|  |  | 177131CLQ |
|  |  | 177105CSI |
|  |  | 495700CSI |
|  |  | 334800CAE |
| Long-acting muscarinic antagonist | Aclidinium | 633730CSI |
|  | Tiotropium | 457301CCH |
|  |  | 457301CPK |
|  |  | 457301CSI |
|  |  | 457330CSI |
|  |  | 503401CSI |
|  |  | 503430CSI |
|  | Umeclidinium | 641101CSI |
| Inhaled corticosteroid | Budesonide | 119402CAE |
|  |  | 119403CAE |
|  |  | 119404CSI |
|  |  | 119407CAE |
|  |  | 119438CAE |
|  |  | 119502CSI |
|  |  | 119504CAE |
|  |  | 119505CSI |
|  |  | 119506CSI |
|  |  | 119530CSI |
|  |  | 119531CSI |
|  |  | 119532CSI |
|  |  | 119533CSI |
|  |  | 391800CSI |
|  |  | 441700CSI |
|  |  | 453400CSI |
|  | Beclomethasone | 502000CSI |
|  |  | 114502CSI |
|  |  | 114503CSI |
|  |  | 114505CCM |
|  |  | 114506CSI |
|  |  | 114508CSI |
|  |  | 114509CSI |
|  |  | 114510CSI |
|  |  | 114530CSI |
|  |  | 114532CSI |
|  |  | 114533CSI |
|  | Ciclesonide | 497101CSI |
|  |  | 497102CSI |
|  |  | 497130CSI |
|  |  | 497131CSI |
|  | Fluticasone | 500431CSI |
|  |  | 500432CSI |
|  |  | 506400CSI |
|  |  | 506500CSI |
|  |  | 506600CSI |
|  |  | 162202CSI |
|  |  | 162203CLQ |
|  |  | 162203CSS |
|  |  | 162204CSI |
|  |  | 162205CSI |
|  |  | 162206CSS |
|  |  | 162230CSS |
|  |  | 162231CSS |
|  |  | 162232CSI |
|  |  | 162233CSI |
|  |  | 162235CSI |
|  |  | 162236CSI |
|  |  | 334500CSI |
|  |  | 334600CSI |
|  |  | 334700CSI |
|  |  | 407100CSI |
|  |  | 407200CSI |
|  |  | 407300CSI |
| Long-acting muscarinic antagonist/long-acting beta-2 agonist | Glycopyrronium/indacaterol | 800100CSI |
|  | Umeclidinium/vilanterol | 631200CSI |
|  | Tiotropium/Olodaterol | 643700CSI |
|  | Aclidinium/formoterol | 635300CSI |
| Inhaled corticosteroid/long-acting beta-2 agonist | Beclometasone/formoterol | 502000CSI |
|  |  | 544200CSI |
|  | Budesonide/formoterol | 543800CSI |
|  |  | 543900CSI |
|  |  | 544000CSI |
|  |  | 544100CSI |
|  |  | 801100CSI |
|  |  | 391800CSI |
|  |  | 441700COS |
|  |  | 441700CSI |
|  |  | 453400CSI |
|  | Fluticasone/salmeterol | 543400CSI |
|  |  | 543500CSI |
|  |  | 543600CSI |
|  |  | 544300CSI |
|  |  | 544400CSI |
|  |  | 544500CSI |
|  |  | 334500CSI |
|  |  | 334600CSI |
|  |  | 334700CSI |
|  |  | 407100CSI |
|  |  | 407200CSI |
|  |  | 407300CSI |
|  |  | 506400CSI |
|  |  | 506500CSI |
|  |  | 506600CSI |
|  |  | 543100CSI |
|  |  | 543200CSI |
|  |  | 543300CSI |
|  | Fluticasone furoate/vilanterol | 636800CSI |
|  |  | 636700CSI |
|  | Fluticasone/formoterol | 544500CSI |
|  |  | 525800CSI |
|  |  | 526200CSI |
|  |  | 542800CSI |
|  |  | 542900CSI |
|  |  | 543000CSI |
| Methylxanthines | Aminophylline | 268500ATB |
|  |  | 386200ATB |
|  |  | 386300ATB |
|  |  | 386500ATB |
|  | Bamiphylline | 113801ATB |
|  | Doxofylline | 439101ATB |
|  | oxtriphylline | 206902AEL |
|  |  | 206903ATB |
|  |  | 206903ATR |
|  | Theophylline | 236901ASY |
|  |  | 237001ACH |
|  |  | 237001ACR |
|  |  | 237001ATB |
|  |  | 237002ACR |
|  |  | 237003ACH |
|  |  | 237003ACR |
|  |  | 237003ASY |
|  |  | 237003ATR |
|  |  | 237003ACR |
|  |  | 237004ACR |
|  |  | 237004ATR |
|  |  | 237005ATR |
|  |  | 237006ATR |
|  |  | 237201ATR |
|  |  | 268800ACH |
|  |  | 374800ASY |
|  |  | 237031ASY |
|  |  | 237003ASY |
| Phosphodiesterase-4 inhibitor | Roflumilast | 614701ATB |

Table S2. Types of proton pump inhibitors

| Medication | Code |
| --- | --- |
| Lansoprazole | 181301ACE |
|  | 181301ATD |
|  | 181302ACE |
|  | 181302ATD |
|  | 181302ATE |
|  | 181301ACH |
|  | 181301ATB |
|  | 181301ATE |
|  | 181302ACH |
| Omeprazole | 204401ACE |
|  | 204401ATE |
|  | 204402ATE |
|  | 204403ATE |
|  | 640200ATB |
|  | 664500ATB |
|  | 204301ACE |
|  | 204301ATB |
|  | 204301ATE |
|  | 204501BIJ |
| Pantoprazole | 208801ATE |
|  | 208802ATE |
|  | 519201ATE |
|  | 519202ATE |
|  | 519203ATE |
|  | 656701ATE |
|  | 208801ATE |
| Rabeprazole | 222201ATE |
|  | 222202ATE |
|  | 222201ATB |
|  | 222202ATB |
| Esomeprazole | 367201ACH |
|  | 367201ATB |
|  | 367202ACH |
|  | 367202ATB |
|  | 498001ACH |
|  | 498002ACH |
|  | 509901ACH |
|  | 509902ACH |
|  | 670700ATB |
|  | 459401BIJ |
| Ilaprazole | 505501ATE |
| Dexlansoprazole | 621901ACR |
|  | 621902ACR |

Table S3. Types of systemic steroid

| Medication | Code |
| --- | --- |
| Deflazacort | 140801ATB |
|  | 140802ATB |
| Dexamethasone | 141901ATB |
|  | 141903ATB |
|  | 141904ATB |
|  | 142030BIJ |
|  | 142201BIJ |
|  | 142202BIJ |
|  | 142230BIJ |
|  | 142232BIJ |
|  | 142233BIJ |
| Hydrocortisone | 170901ATB |
|  | 170905ATB |
|  | 170906ATB |
|  | 171201BIJ |
|  | 171202BIJ |
|  | 171203BIJ |
| Methylprednisolone | 193301ATB |
|  | 193302ATB |
|  | 193304ATB |
|  | 193305ATB |
|  | 193501BIJ |
|  | 193502BIJ |
|  | 193530BIJ |
|  | 193531BIJ |
|  | 193601BIJ |
|  | 193602BIJ |
|  | 193603BIJ |
|  | 193604BIJ |
| Prednisolone | 217001ATB |
|  | 217003ASY |
|  | 217004ASY |
|  | 217030ASY |
|  | 217034ASY |
|  | 217035ASY |
|  | 217104BIJ |
|  | 217301BIJ |
|  | 217302BIJ |
| Triamcinolone | 243201ATB |
|  | 243202ATB |
|  | 243203ATB |
|  | 243301BIJ |
|  | 243303BIJ |
|  | 243305BIJ |
|  | 243335BIJ |
|  | 243336BIJ |
|  | 243337BIJ |
| Betamethasone | 296900ATB |
|  | 116401ATB |
|  | 116501ATB |
|  | 116502BIJ |
|  | 116530BIJ |

Table S4. Types of antibiotics

| Class | Medication | Code |
| --- | --- | --- |
| Penicillin | Penicillin | 210001BIJ |
|  |  | 115502BIJ |
|  |  | 115501ATB |
|  |  | 210002BIJ |
|  |  | 210101BIJ |
|  |  | 210102BIJ |
|  | Nafcillin | 360801BIJ |
|  | Amoxicillin | 108101ACH |
|  |  | 108103ACH |
|  |  | 108130ASY |
|  |  | 108201BIJ |
|  |  | 108203BIJ |
|  |  | 108101ATB |
|  |  | 108102ASY |
|  |  | 108103ATB |
|  |  | 108202BIJ |
|  | Ampicillin | 589301ACH |
|  |  | 589302ACH |
|  |  | 108603BIJ |
|  |  | 108601ACH |
|  |  | 108601BIJ |
|  |  | 108602ASY |
|  |  | 108603ACH |
|  |  | 108604ASY |
|  | Piperacillin | 213104BIJ |
|  |  | 433300BIJ |
|  |  | 453200BIJ |
|  |  | 657600BIJ |
|  |  | 213101BIJ |
|  |  | 213102BIJ |
|  |  | 213103BIJ |
|  | Piperacillin/tazobactam | 329500BIJ |
|  |  | 329600BIJ |
|  | Ampicillin/sulbactam | 328600BIJ |
|  |  | 328500BIJ |
|  | Amoxicillin/sulbactam | 381300BIJ |
|  |  | 381500BIJ |
|  |  | 380000ATB |
|  |  | 536200ASY |
|  |  | 536300ASY |
|  |  | 381400BIJ |
|  | Amoxicillin/clavulanate | 328900BIJ |
|  |  | 329000BIJ |
|  |  | 440100ATB |
|  |  | 310500ATB |
|  |  | 310600ATB |
|  |  | 310700ATB |
|  |  | 462000ATB |
|  |  | 467300ASS |
|  |  | 467400ASS |
|  |  | 534200ASY |
|  |  | 534400ASY |
|  |  | 534600ASY |
|  |  | 534700ASY |
|  |  | 534800ASY |
|  |  | 535000ASY |
|  |  | 535300ASY |
|  |  | 535500ASY |
|  |  | 536300ASY |
|  |  | 647300ASY |
|  |  | 358500ASY |
|  |  | 379800ASY |
|  |  | 379900ASY |
|  |  | 467200ATB |
|  |  | 467600ATB |
|  |  | 504000ASY |
|  |  | 504100ASY |
|  |  | 534100ASY |
|  |  | 534300ASY |
|  |  | 534500ASY |
|  |  | 534900ASY |
|  |  | 535100ASY |
|  |  | 535200ASY |
|  |  | 535400ASY |
|  |  | 535600ASY |
|  |  | 535700ASY |
|  |  | 535800ASY |
|  |  | 536100ASY |
|  |  | 536200ASY |
|  | Ticarcillin | 329700BIJ |
| Cephalosporin (2^nd^ generation) | Cefoxitin | 127501BIJ |
|  |  | 127502BIJ |
|  | Cefprozil | 128001ATB |
|  |  | 128030ASY |
|  |  | 128031ASY |
|  |  | 128032ASY |
|  |  | 128002ASY |
|  | Cefuroxime | 128903ATB |
|  |  | 128931ASY |
|  |  | 129001BIJ |
|  |  | 129002BIJ |
|  |  | 129003BIJ |
|  |  | 128901ATB |
|  |  | 128902ASY |
|  |  | 128904ATB |
|  |  | 128930ASY |
|  |  | 128932ASY |
|  | Cefmetazole | 126501BIJ |
|  |  | 126502BIJ |
|  |  | 126503BIJ |
|  |  | 468100BIJ |
|  | Cefaclor | 125201ACH |
|  |  | 125204ATR |
|  |  | 125232ASY |
|  |  | 125235ASY |
|  |  | 125237ASY |
|  |  | 125238ASY |
|  |  | 125201AGN |
|  |  | 125202ASY |
|  |  | 125203ASY |
|  |  | 125204ACH |
|  |  | 125205ACH |
|  |  | 125206ASY |
|  |  | 125208ASY |
|  |  | 125209AGN |
|  |  | 125210ATB |
|  |  | 125230AGN |
|  |  | 125233ASY |
|  |  | 125234ASY |
|  |  | 125236ASY |
|  |  | 125239ASY |
|  |  | 125240AGN |
|  | Cefotetan | 127201BIJ |
|  |  | 127203BIJ |
|  |  | 482900BIJ |
|  |  | 127202BIJ |
|  | Cefamandole | 125401BIJ |
|  |  | 125402BIJ |
|  |  | 125501BIJ |
|  |  | 125502BIJ |
|  | Cefbuperazone | 125801BIJ |
|  |  | 125802BIJ |
|  | Cefminox | 126601BIJ |
|  |  | 126602BIJ |
| Cephalosporin (3^rd^ generation) | Cefotiam | 127301BIJ |
|  |  | 127302BIJ |
|  |  | 127303BIJ |
|  |  | 465400BIJ |
|  |  | 127401ATB |
|  |  | 127402ATB |
|  | Cefotaxime | 127101BIJ |
|  |  | 127102BIJ |
|  |  | 127103BIJ |
|  |  | 127104BIJ |
|  | Cefdinir | 125901ACH |
|  |  | 125931AGN |
|  |  | 125901AGN |
|  |  | 125902ACH |
|  |  | 125930AGN |
|  |  | 125932AGN |
|  | Ceftibuten | 128602ACH |
|  |  | 128630ASY |
|  |  | 128601ASY |
|  |  | 128603ACH |
|  |  | 128604ACH |
|  |  | 128631ASY |
|  | Cefodizime | 126701BIJ |
|  |  | 126702BIJ |
|  | Cefditoren | 126001ATB |
|  |  | 126031AGN |
|  |  | 126001AGN |
|  |  | 126030AGN |
|  | Ceftriaxone | 128801BIJ |
|  |  | 128802BIJ |
|  |  | 128803BIJ |
|  |  | 128804BIJ |
|  |  | 128805BIJ |
|  |  | 468200BIJ |
|  |  | 478500BIJ |
|  | Cefpodoxime | 127901ATB |
|  |  | 127930ASY |
|  |  | 127931ASY |
|  |  | 127932ASY |
|  |  | 127903ASY |
|  | Ceftazidime | 128301BIJ |
|  |  | 128302BIJ |
|  |  | 128303BIJ |
|  | Cefixime | 126301ACH |
|  |  | 126334APD |
|  |  | 126302ACH |
|  |  | 126302APD |
|  |  | 126330APD |
|  |  | 126331APD |
|  |  | 126332APD |
|  |  | 126333APD |
|  | Cefetamet | 126201ATB |
|  |  | 126202APD |
|  |  | 126202ATB |
|  | Ceftizoxime | 128701BIJ |
|  |  | 128702BIJ |
|  | Cefoperazone | 329900BIJ |
|  |  | 463100BIJ |
|  |  | 557400BIJ |
|  |  | 126901BIJ |
|  |  | 126902BIJ |
|  |  | 126903BIJ |
|  |  | 371300BIJ |
|  | Cefcapene | 474401ATB |
|  |  | 474402ATB |
|  |  | 474432AGN |
|  |  | 474430AGN |
|  |  | 474431AGN |
|  |  | 474432AGN |
|  | Cefpiramide | 127701BIJ |
|  |  | 127702BIJ |
|  | Cefodizime | 126701BIJ |
|  |  | 126702BIJ |
|  | Flomoxef | 159501BIJ |
| Cephalosporin (4^th^ generation) | Cefepime | 126101BIJ |
|  |  | 126103BIJ |
|  |  | 800001BIJ |
|  |  | 126102BIJ |
|  | Cefpirome | 127801BIJ |
|  |  | 127802BIJ |
| Monobactam | Aztreonam | 113001BIJ |
|  |  | 113002BIJ |
| Fluoroquinolone | Levofloxacin | 183201ATB |
|  |  | 183202ATB |
|  |  | 183203ATB |
|  |  | 183205ATB |
|  |  | 183233BIJ |
|  |  | 183234BIJ |
|  |  | 183235BIJ |
|  |  | 183236BIJ |
|  |  | 183202BIJ |
|  |  | 183203BIJ |
|  |  | 183205BIJ |
|  |  | 183206BIJ |
|  | Moxifloxacin | 380301ATB |
|  |  | 380335BIJ |
|  |  | 801601BIJ |
|  |  | 380302BIJ |
|  |  | 380303BIJ |
|  |  | 434801ATB |
|  | Ciprofloxacin | 134103ATB |
|  |  | 134105ATB |
|  |  | 134108ATR |
|  |  | 134109ATB |
|  |  | 134133BIJ |
|  |  | 134134BIJ |
|  |  | 134135BIJ |
|  |  | 134101ATB |
|  |  | 134101BIJ |
|  |  | 134103ACH |
|  |  | 134104BIJ |
|  |  | 134105ATR |
|  |  | 134106BIJ |
|  | Gemifloxacin | 442901ATB |
|  |  | 442902BIJ |
| Macrolide | Azithromycin | 112701ATB |
|  |  | 112705ATB |
|  |  | 112732ASY |
|  |  | 112733ASY |
|  |  | 112734BIJ |
|  |  | 112731ASY |
|  |  | 439901BIJ |
|  |  | 112701ACH |
|  |  | 112702ASY |
|  | Clarithromycin | 134901ATB |
|  |  | 134904ATB |
|  |  | 134904ATR |
|  |  | 134933ASY |
|  |  | 134934ASY |
|  |  | 134935ASY |
|  |  | 134942ASY |
|  |  | 134936ASY |
|  |  | 134937ASY |
|  |  | 134939ASY |
|  |  | 134902BIJ |
|  |  | 134903ASY |
|  |  | 134905ASY |
|  |  | 134930ASY |
|  |  | 134931ASY |
|  |  | 134938ASY |
|  |  | 134940ASY |
|  |  | 134941ASY |
|  | Roxithromycin | 225301ATB |
|  |  | 225302ASS |
|  |  | 225302ATB |
|  |  | 225332ASS |
|  |  | 225333AGN |
|  |  | 225337AGN |
|  |  | 225302AGN |
|  |  | 225304ASS |
|  |  | 225330AGN |
|  |  | 225331AGN |
|  |  | 225334AGN |
|  |  | 225335AGN |
|  |  | 225336AGN |
| Carbapenem | Imipenem | 329300BIJ |
|  |  | 329400BIJ |
|  |  | 466100BIJ |
|  | Ertapenem | 447701BIJ |
|  | Meropenem | 190702BIJ |
|  |  | 190703BIJ |
|  |  | 190704BIJ |
|  |  | 190701BIJ |
|  | Doripenem | 593201BIJ |
| Glycopeptides | Vancomycin | 247202ACH |
|  |  | 247203BIJ |
|  |  | 247204BIJ |
|  |  | 247205BIJ |
|  |  | 479800BIJ |
|  |  | 247201BIJ |
|  | Teicoplanin | 234901BIJ |
|  |  | 234902BIJ |
|  |  | 501000BIJ |
| Polymyxin E | Colistin | 484201BIJ |
|  |  | 484202BIJ |
|  |  | 484203BIJ |
